# Chest wall perforator flaps in partial breast reconstruction after breast conservation surgery: an additional oncoplastic surgical option

**DOI:** 10.3332/ecancer.2020.1073

**Published:** 2020-07-17

**Authors:** Sanjit Kumar Agrawal, Sudip Ratna Shakya, Shashank Nigam, Abhishek Sharma, Soumitra S Datta, Rosina Ahmed

**Affiliations:** 1 Department of Breast Oncosurgery, Tata Medical Center, Kolkata 700156, India; 2 Department of Palliative Care and Psycho-Oncology, Tata Medical Center, Kolkata 700156, India; 3 MRC Clinical Trials Unit, Institute of Clinical Trials & Methodology, University College London, WC1V 6LJ, United Kingdom

**Keywords:** breast cancer, oncoplastic breast surgery, chest wall perforator flap

## Abstract

Partial breast reconstruction using chest wall perforator flaps (CWPF) is a recent option used by breast surgeons, mainly for lateral quadrant defects with a relatively large volume of excision. We report a single-centre experience of CWPF with surgery details, complications, re-excision, aesthetic and oncological outcomes.

This was a prospective observational cohort study of patients who had undergone breast conservation surgery (BCS) plus CWPF reconstruction. All variables were recorded prospectively in the institutional database. A survey was done to analyse patient satisfaction at about 6 months after completion of radiotherapy.

Forty patients had CWPF based reconstruction in 3 years. 57.5 % of patients had lateral intercostal artery perforator (LICAP) flap, 5% had lateral thoracic artery perforator (LTAP) flap, 27.5% had combined LICAP plus LTAP and 10% patients had anterior intercostal artery perforator (AICAP) flap. Tumour excision cavity defect was of the lateral quadrant in 82.5%, central quadrant in 10% and medial quadrant in 7.5% of patients. The margin was positive for five patients, out of which four required cavity shave and one had a mastectomy. One patient had complete flap loss, and two patients developed surgical site infection. 96% of patients were satisfied with the scar, and 88% were happy with the treated breast in comparison to the opposite breast. 92% were comfortable going out in public and felt that in retrospect their decision not to have a mastectomy was correct. With a median follow up of 18 (10, 22) months, one patient died, and four had recurrences.

CWPF may be used for partial breast reconstruction in the small non-ptotic breast with excellent outcome and high patient satisfaction scores.

## Background

Breast conservation surgery (BCS) with whole-breast irradiation is well established in the management of early breast cancer. It is equivalent to mastectomy in terms of survival and local control, with the added advantage of achieving an excellent cosmetic outcome, high patient satisfaction and improved quality of life in all the age groups [1–3]. Oncoplastic breast surgery (OBS) adds to cosmetic outcomes, and many new surgical techniques have emerged and evolved to facilitate partial breast reconstruction. The development of oncoplastic surgical methods in the last decade has increased the BCS rates globally and enabled improved cosmetic outcomes without compromising oncological safety [4].

For patients with small to moderate-sized breasts and large tumours, the removal of the mass alone may leave an unacceptably large defect. In such cases, some forms of oncoplastic volume replacement techniques are required to achieve breast symmetry. At present, there is no consensus on which OBS technique should be used for any given situation, and it is usually the tumour board (including breast and plastic surgeons) that chooses the reconstruction options based on the experience, tumour to breast size ratio etc. For many years, Latissimus Dorsi (LD) myocutaneous/mini LD flaps were used to cover large defects in small breasts with the disadvantage of donor site morbidity and functional impairment of the shoulder [5]. Chest wall perforator flap (CWPF)-based reconstruction has recently emerged as a valuable option in this particular scenario (small to medium-sized breasts, with the large tumours).

In 2005, Hamdi et al [6, 7] reported the use of CWPF in the form of thoracodorsal artery perforator (TDAP) and intercostal artery perforator (ICAP) flaps. They showed the safety and feasibility of CWPF in breast reconstruction. In 2014, McCulley et al [8] introduced the lateral thoracic artery perforator (LTAP) flap as an additional option for the reconstruction of partial breast defects [8]. These techniques offer an excellent opportunity for partial breast reconstruction in women with small-to-moderate-size non-ptotic breast and may prevent the need for mastectomy or LD flap harvest in a selected group of patients who wish to undergo BCS. CWPF is often criticised for the long chest wall scar that it creates, but mostly this scar gets hidden in the bra line. However, patient satisfaction with the scar needs to be assessed. Recently, authors have reported that besides functional benefits, CWPFs have the added advantage of minimal donor site morbidity with excellent cosmetic outcomes and better patient satisfaction [6–8].

BCS with CWPF reconstruction was started at Tata Medical Center in 2017 and is increasingly being offered to patients with small-to-moderate-sized breasts with large defects. The outcome of CWPF-based breast reconstruction in the Asian woman is mostly unknown. In this study, we share our experience with the use of CWPF breast reconstruction following BCS, with regards to details of surgical procedure, complications, cosmesis and patient satisfaction.

## Methods

All women who underwent BCS and partial breast reconstruction with CWPF for breast cancer from January 2017 to November 2019 at Tata Medical Center, Kolkata, India were included in this study (ethics committee waiver No: EC/WV/TMC/014/19). Data on clinicopathological characteristics, surgical details, adjuvant treatment and follow-up were retrieved from the institutional hospital management system, and a prospectively maintained REDCap database.

For all patients, tumour excision and CWPF reconstruction were done at the same time as a single procedure. All patients were marked before the surgery in the standing position. The breast band size and cup size were measured preoperatively and recorded in the database. Chest wall perforators were marked on the skin using a handheld Doppler ultrasound probe. Flaps were marked to include the perforators, based on the tumour size and the estimated location of the defect. The axillary staging was done using the same incision planned for raising the flap. In the majority of cases, wide local excision was also done through the planned flap incision. After wide local excision, the flap was raised in the lateral to medial direction, and perforators were identified and preserved. Intraoperative Doppler USG was used to confirm perforator patency and position. The flap was rotated on the perforators to fill the breast defect, making sure not to twist or kink the perforators. After an episode of complete flap loss, we have started the use of intra-operative flap monitoring using indocyanine green (ICG) dye. The adequacy of flap vascularisation was confirmed with an infrared enabled ICG probe. Following institutional protocols, no ink on the tumour margin was considered as a negative margin for invasive cancer and 2 mm for pure ductal carcinoma in situ (DCIS). All patients with positive margins on surgical specimen histopathology report had a subsequent cavity excision to ensure clear margins. All patients received adjuvant treatment as per institutional MDT decisions.

An acquired-informal questionnaire was developed and used to assess patient satisfaction at least 6 months after the completion of radiotherapy, using a 4-point Likert’s scale. The survey was administered either telephonically or during a routine follow-up visit. Patient permission was obtained for anonymised use of photographs as per institutional protocols. Summary statistics (median, interquartile range and percentage) was used for the data analysis using SPSS 23.

## Results

Between January 2017 and November 2019, 40 patients underwent CWPF for partial breast reconstruction. Out of the 40 cases, 32 (80%) patients had upfront surgery and 8 (20%) patients had surgery post neoadjuvant chemotherapy. The median age was 44 years (IQR 39,48), and median BMI was 25.46 kg/m^2^ (IQR 22.97, 27.70). The median waiting time from diagnosis to surgery for upfront cases was 18 days (IQR 16,25). All patients were discharged within 24 hours following the surgical procedure. The median follow-up was 18 (IQR 10, 22) months. Median breast band size was 34 (IQR 32,36), and the majority of patients had breasts of B (30%) or C (52.5%) cup size.

Lateral intercostal artery perforator (LICAP), anterior intercostal artery perforator (AICAP) and LTAP flap were performed in 23 (57.5%), 4 (10%) and 2 (5%) patients, respectively. 11 (27.5%) patients had combined LTAP and LICAP. Lateral quadrant defects (33/40) were reconstructed by LICAP/LTAP flap or combination of two, and medial quadrant defects (4/40) by AICAP flap. Three patients had a lateral tumour with nipple-areola excision, which was reconstructed by using LICAP in two patients and LICAP plus LTAP in one patient. Surgical details and histopathological characters are summarised in Table 1.

One patient had total flap loss, which was managed by debridement, and the defect was covered with a mini LD flap. Two patients developed surgical site infections (SSI) which were managed conservatively. Five (12.8%) patients had positive margins, out of which four required cavity shave and one underwent completion mastectomy because of extensive DCIS and patient choice. Seroma was not reported in any patient. Thirty eight patients are under regular follow up. At a median follow up of 18 months, four patients had developed distant metastatic disease, one had an axillary recurrence (post-sentinel lymph node biopsy) and one was lost to follow up. There was only one mortality in the series, in a patient who died of sepsis following her sixth cycle of chemotherapy. There were no local recurrences.

Of 35 patients who were at least 6 months post-radiotherapy, 25 (71%) responded to the follow-up survey questionnaire. 96% were satisfied with the scar, and 88% were happy with the treated breast in comparison to the opposite breast. 92% were comfortable going out in public places and felt that in retrospect their decision not to have a mastectomy was correct (Table 2).

## Discussion

This study showed that CWPFs offer an excellent option for partial breast reconstruction in women with small and medium-sized breasts, with good patient-reported aesthetic outcomes and minimal morbidity. Various CWPFs used in partial breast reconstruction are reported in the literature, including TDAP, LTAP, LICAP and AICAP [6–9]. Apart from TDAP, all of these flaps were used in our series. We found that CWPFs could be used for tumours located in any quadrant of the breast although they were most frequently used laterally, and rarely in the upper inner quadrant.

**Figure 1.**
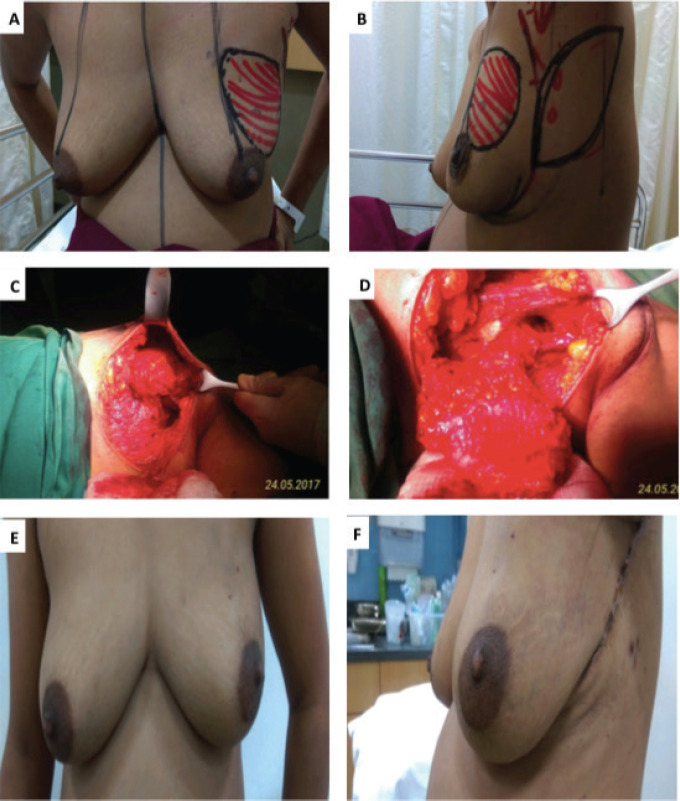
A.B. Preoperative marking and localization of perforators for LICAP; C,D. The tumour was excised and the flap was completely isolated on one lateral perforators; E, F. Post operative pictures

Hamdi [7, 9] reported the first use of LICAP and AICAP flaps in partial breast reconstruction. The LICAP flap is based on perforators originating from the intercostal segment of the intercostal vessels, which are commonly found in the 5th to 7th intercostal spaces between 2.5 and 3.5 cm medial to the anterior border of the LD muscle [10]. The LICAP flap is most suitable for defects in the lateral quadrant of the breast. In our series too, the LICAP flap was used most frequently, in 23 patients, all of whom had tumours located in the lateral quadrant of the breast (Figure 1—superolateral quadrant tumour excision with LICAP flap).

**Figure 2.**
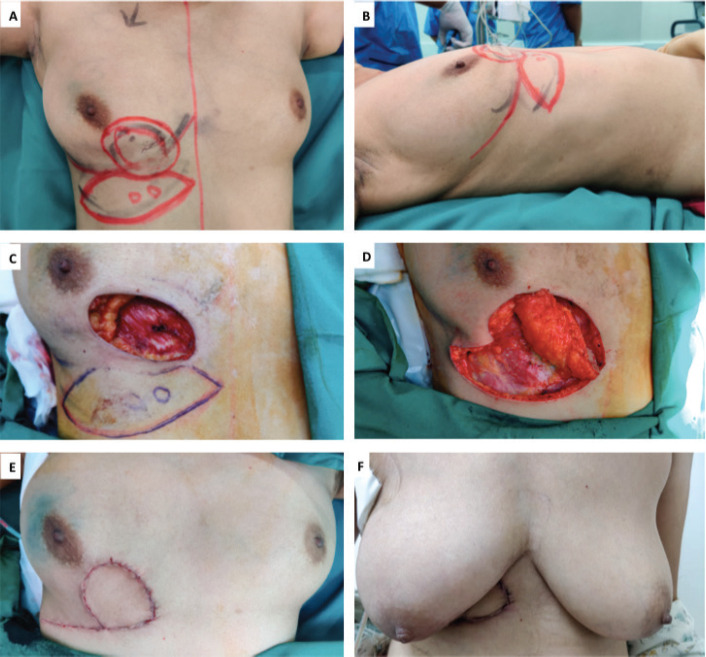
A,B. Preoperative marking and localization of perforators for AICAP; C,D. The tumour was excised and the flap was completely isolated on anterior intercostal perforators; E. Immediate Post operative picture; F. On follow up.

The AICAP flap is based on perforators originating from the muscular or rectal segment of the intercostal vessels, which are located within 1–3 cm lateral to the sternal border [9]. The flap is suitable for defects in the medial aspect of the breast. In our series, it was mainly used to fill defects in the inferomedial quadrant (Figure 2—Inferomedial quadrant tumour excision with AICAP flap).

**Figure 3.**
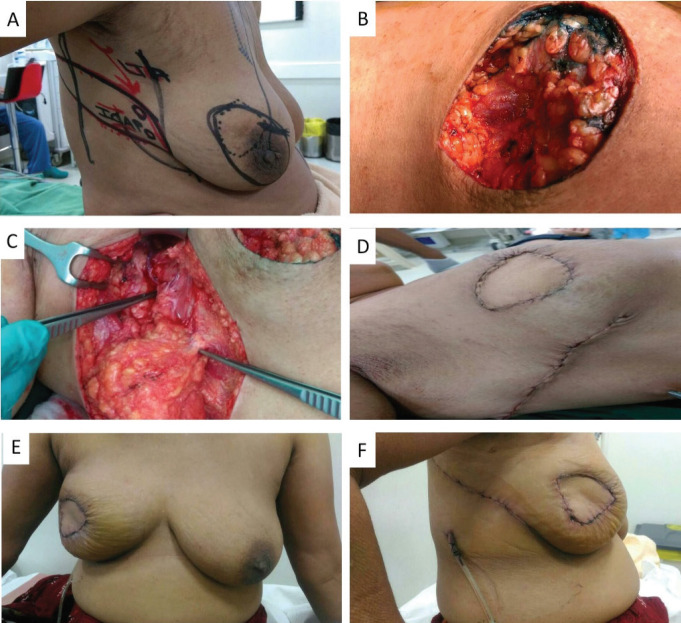
A. Preoperative marking and localization of perfortors for combined LTAP plus ICAP; B. Defect after tumour excision; C. The flap was completely isolated on the perforators; D: Immediate post op pictures; E, F. On follow up. Patient was not ready for contralateral symmetrisarion.

The LTAP flap for partial breast reconstruction was first described McCulley [8] and is based on single or multiple perforators of the lateral thoracic vessels. LTAP perforators are found in the 3rd and 4th intercostal spaces within 2 cm of the lateral breast crease. The LTAP flap is suitable for defects in the lateral aspect of the breast, and it can be partly or wholly mobilised to allow greater reach and transposition compared to the LICAP flap. The LTAP flap can be used either alone as the main pedicle, or in combination with the LICAP, augmenting perfusion to the skin paddle. In our series, LTAP flap was used solely for 2 (5%), and a combination of LTAP with LICAP flap was used in 11 (27.5%) patients (Figure 3—Retroareolar mass excision with combined LTAP plus LICAP flap). As these perforators are based on the intercostal vessels with the intact thoracodorsal pedicle, it gives surgeons an additional option of LD flap to cover the defects in situations of reoperation due to margin positivity or flap loss.

One of the significant challenges in the use of CWPF is reoperation for positive margins. Reoperation may have an adverse effect on the cosmetic outcome and may compromise patient satisfaction. In our series, the margin was positive in five (12.5%) patients. This is comparable to other studies, which have reported positive margins in about 10% of cases (Table 3).

We follow the Society of Surgical Oncology (SSO) and American Society for Radiation Oncology (ASTRO) consensus guidelines, which standardise a negative margin after BCS as having ‘no ink on tumour’ in patients with invasive cancer [11]. In our series, of the five cases with positive margins, four were involved by DCIS and one by the invasive tumour. Rosenberger et al [15] recently reported their early experience following the changes in the SSO/ASTRO guidelines. In this study, the authors found that the disease present at the margin of excision in almost half the patients was DCIS, with the remainder having invasive cancer.

Intraoperative assessment of the surgical margin status using frozen section is an option which is used in some centres. However, the frozen section is time-consuming and does not guarantee complete excision of the tumour, and there is always the possibility of having positive margins reported on the final histopathological report. In a study by Munhoz *et al* [16], 218 patients underwent BCS with reconstruction and had frozen section to confirm excision margins, but final histopathology reports showed positive margins in 5.5% of these patients in spite of a previously negative margin reported on the frozen section. In our institution, the frozen section is not used routinely for margin assessment although it is available for use if required. In this series, the frozen section was not requested for any of the patients.

Another way to avoid the consequences of positive margins is to perform the procedure as a 2-stage approach. Roy *et al* [13] recently showed that the two-stage method had excellent outcomes with high patient satisfaction. The two-stage process involves initial lumpectomy with an axillary procedure as indicated, followed by reconstructive surgery within 2–3 weeks once final histopathology has confirmed negative margins. However, in the resource-constrained country, patients are often reluctant to have multiple surgeries, and two-stage procedures are generally not well accepted by patients. In our series, all patients were offered excision and CWPF reconstruction as a single-stage procedure.

Flap related complications may have an adverse effect on cosmetic outcomes and are reported in up to 5%–10% of cases. ICG dye-based fluorescent angiography is a useful technique for the assessment of blood flow and tissue perfusion in skin flaps [17]. Several studies have shown that intraoperative perfusion mapping of flaps with ICG can predict and prevent complications in breast reconstructive surgery [18, 19]. After one of our patients had flap necrosis, we started to assess flap perfusion routinely, using ICG. We especially found it a useful technique in cases with small perforators to evaluate the vascularity of flap intraoperatively (Figure 3—Flap vascularity assessment using fluorescent dye). The additional cost of ICG use in our set up was 20 USD only per patient, which was <1% of total surgery expense.

In the patient satisfaction survey, which was conducted 6 months after the completion of radiotherapy, our results were similar to those reported in a similar study done in Korean women [12]. Most of the patients in our series had small to medium-sized breasts, and they benefited from the use of CWPF, with good oncological outcomes and reasonable patient satisfaction. We have summarised studies published, which describe the use of CWPF, in Table 3. All of these studies have shown acceptable surgical complication rates (around 10%) and high satisfaction among patients (90%), and our study also had similar findings.

This study has some potential limitations. Patient satisfaction was assessed with Likert’s scale and needed a more robust validated method like body image scale or Breast Q [20, 21]. In the present study, the frozen section was not used to assess the margin status. This could be potentially explored in a future series to reduce the reoperation rate. Those patients with minor contour deformities could have benefitted from additional procedures like deformity correction, lipomodelling and contralateral symmetrisation. However, in our resource-constrained setup, where the patients and their immediate family members support the treatment costs mostly from out of pocket expenses, the demand for secondary corrections is negligible. As a result, no additional cosmetic procedure has been done in our cohort. Another limitation was the small sample size and short follow-up duration of 18 months. Long-term results from large series are needed to assess the cosmetic and survival outcome of the CWPF-based reconstruction.

## Conclusion

CWPF procedures show good outcomes in partial breast reconstruction in terms of oncological safety and patient satisfaction. Our study has shown that this technique may be used for tumours located in lateral, central and inferomedial quadrants of the breast. Proper patient selection and good preoperative planning and design of flaps are needed to achieve a good outcome. Careful dissection of the flap and confirmation of the vascularity of the raised flap is helpful if the technology is available, using intraoperative Doppler and infrared fluorescence system.

## Abbreviations

- **ASTRO:** American Society for Radiation Oncology
- **AICAP:** anterior intercostal artery perforator
- **BCS:** breast conservation surgery
- **CWPF:** chest wall perforator flap
- **DCIS:** ductal carcinoma in situ
- **ICG:** indocyanine green
- **ICAP:** intercostal artery perforator
- **LICAP:** lateral intercostal artery perforator
- **LTAP:** lateral thoracic artery perforator
- **LD:** latissimus dorsi
- **MS:** muscle sparing
- **NR:** not reported
- **OBS:** oncoplastic breast surgery
- **SSO:** Society of Surgical Oncology
- **SSI:** surgical site infections
- **TDAP:** thoracodorsal artery perforator

## Authors’ contributions

Conceptualisation (Sanjit Kumar Agrawal), Methodology (Sanjit Kumar Agrawal, Soumitra Shankar Datta, Rosina Ahmed), Data Collection (Sudip Ratna Shakya, Shashank Nigam), Data Analysis (Sanjit Kumar Agrawal, Sudip Ratna Shakya), Writing—Original Draft Preparation (Sanjit Kumar Agrawal, Sudip Ratna Shakya), Writing—Review and editing ( All authors), Final approval of manuscript (All authors).

## Declaration of conflicts of interest

The authors declare no conflicts of interest.

## Funding disclosure

There was no funding received for this study.

## Figures and Tables

**Figure 4.**
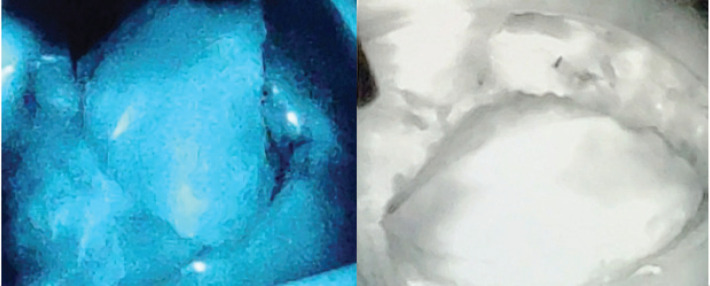
Indocyanine green (ICG) immunofluorescence intraoperative screen of an AICAP flap. The flap is seen well-perfused.

**Table 1.** Surgical and histopathological details.

	N (%)/ Median (IQR)
Axillary surgery Sentinel lymph node dissection (SLND) Axillary lymph node dissection (ALND) None	14 (35%) 25 (62.5%) 1 (2.5%)
Tumour location Superolateral Superomedial Inferolateral Inferomedial Central	29 (72.5%) 1 (2.5%) 4 (10%) 3 (7.5%) 3 (7.5%)
Pathological tumour size Length (cm) Width (cm)	2.6 (2.0, 3.0) 1.8 (1.5, 2.0)
Flap dimension Length (cm) Width (cm)	14.0 (12.0-15.0) 8.0 (7.0-9.0)
Flap positioning Including skin Fully de-epithelialised	19 (47.5%) 21 (52.5%)
Specimen weight (grams)	120 (109, 157)
Operation time (min)	180 (144, 190)
Type of carcinoma Infiltrating ductal carcinoma Infiltrating mucinous carcinoma	37 (92.5%) 3 (7.5%)
Tumour Grade 1 2 3	2 (5%) 13 (32.5%) 25 (62.5%)
T stage T1 T2 T3	8 (20%) 29 (72.5%) 3 (7.5%)
N stage N0 N1 N2 N3 Nx	20 (50%) 9 (22.5%) 8 (20%) 2 (5%) 1 (2.5%)
TNM stage I II III	6 (15%) 24 (60%) 10 (25%)
Tumour subtype Luminal A Luminal B HER 2 enriched Triple-negative	13 (32.5%) 17 (42.5%) 3 (7.5%) 7 (17.5%)

**Table 2.** Patient satisfaction assessment.

	Highly unsatisfied	Unsatisfied	Satisfied	Highly satisfied
How satisfied are you with your scar?	0 (0%)	1 (4%)	15 (60%)	9 (36%)
How happy are you with your treated breast in comparison to the opposite breast?	0 (0%)	3(12%)	9 (39%)	13 (52%)
How comfortable are you going out in public?	0 (0%)	2 (8%)	12 (48%)	11 (44%)
Do you feel, in retrospect, that you should have opted for a mastectomy?	0 (0%)	2 (8%)	2 (8%)	19 (84%)

**Table 3.** Published outcome data of CWPF in partial breast reconstruction.

	Year, location	*n*	CWPF used	Margin positivity	Complications	Satisfaction
Present cohort	2019, India	40	AICAP LICAP LTAP	5 (12.5%)	Total flap loss 1 (2.5%) SSI 2 (5%)	Excellent/good (95%)
Kim JB *et al* [12]	2017, Korea	33	TDAP LICAP	3 (9.1%)	Wound disruption 4 Linear necrosis 8 Fat necrosis 4	Excellent/good (84.8%)
Roy PK [13]	2016, UK	40	LTAP LICAP	4 (10%)	Superficial necrosis 1 Fat necrosis 2	Excellent/good (82%)
McCulley *et al* [8]	2015, UK	75	LTAP LICAP	0	Superficial necrosis 1 Fat necrosis 2	NR
Munhoz *et al* [14]	2011, Brazil	13	LICAP	0	Wound dehiscence (15.3%) Fat necrosis 1 (7.6%)	Satisfied (90%)
Hamdi *et al* [9]	2006, Belgium	16	LICAP	NR	Wound dehiscence (12.5%)	NR
Hamdi *et al* [6]	2004, Belgium	31	TDAP LICAP MS LD	NR	Seroma (12.9%) Wound dehiscence: (6.4%) Partial necrosis (9.6%)	NR

NR, Not Reported; MS, Muscle Sparing.
